# Study on the Mechanism of Baimai Ointment in the Treatment of Osteoarthritis Based on Network Pharmacology and Molecular Docking with Experimental Verification

**DOI:** 10.3389/fgene.2021.750681

**Published:** 2021-11-17

**Authors:** Yingyin Zhu, Wanling Zhong, Jing Peng, Huichao Wu, Shouying Du

**Affiliations:** ^1^ School of Chinese Materia Medica, Beijing University of Chinese Medicine, Beijing, China; ^2^ School of Traditional Chinese Medicine, Beijing University of Chinese Medicine, Beijing, China

**Keywords:** Baimai ointment, osteoarthritis, network pharmacology, molecular docking, molecular mechanism, external preparation

## Abstract

**Purpose:** The external preparation of the Tibetan medicine formula, Baimai ointment (BMO), has great therapeutic effects on osteoarthritis (OA). However, its molecular mechanism remains almost elusive. Here, a comprehensive strategy combining network pharmacology and molecular docking with pharmacological experiments was adopted to reveal the molecular mechanism of BMO against OA.

**Methods:** The traditional Chinese medicine for systems pharmacology (TCMSP) database and analysis platform, traditional Chinese medicine integrated database (TCMID), GeneCards database, and DisGeNET database were used to screen the active components and targets of BMO in treating OA. A component–target (C-T) network was built with the help of Cytoscape, and the Gene Ontology and Kyoto Encyclopedia of Genes and Genomes (KEGG) pathway enrichment through STRING. Autodock Tools which was used to dock the key components and key target proteins was analyzed. Animal experiments were performed to verify the key targets of BMO. Hematoxylin–eosin and toluidine blue staining were used to observe the pathology of joints. Protein expression was determined using enzyme-linked immunosorbent assay.

**Results:** Bioactive compounds and targets of BMO and OA were screened. The network analysis revealed that 17-β-estradiol, curcumin, licochalone A, quercetin, and glycyrrhizic acid were the candidate key components, and IL6, tumor necrosis factor (TNF), MAPK1, VEGFA, CXCL8, and IL1B were the candidate key targets in treating OA. The KEGG indicated that the TNF signaling pathway, NF-κB signaling pathway, and HIF-1 signaling pathway were the potential pathways. Molecular docking implied a strong combination between key components and key targets. The pathology and animal experiments showed BMO had great effects on OA *via* regulating IL6, TNF, MAPK1, VEGFA, CXCL8, and IL1B targets. These findings were consistent with the results obtained from the network pharmacology approach.

**Conclusion:** This study preliminarily illustrated the candidate key components, key targets, and potential pathways of BMO against OA. It also provided a promising method to study the Tibetan medicine formula or external preparations.

## Introduction

Osteoarthritis (OA) is a common chronic disease with cartilage degradation in the clinic, characterized by arthritis, synovial inflammation, joint edge, and underlying subchondral bone lesions (Krustev et al., 2015). Clinical manifestations include joint pain, swelling, stiffness, and even deformity with limited movement (Nüesch et al., 2011; Centers for Disease Control and Prevention, 2020). At present, the treatment of OA mainly includes oral drug therapy, intra-articular injection, exercise, and surgery (Sellam et al., 2019; Tao et al., 2020), which was reported to have the limitations of drug tolerability and side effects, inadequate symptom control, and invasive intervention (Xu et al., 2017; Kolasinski et al., 2020). Therefore, a noninvasive and effective treatment with better bioavailability and less systemic toxicity needs to be developed.

Tibetan medicine, the second largest traditional Chinese medicine system in China, has rich clinical applications in treating OA with its characteristic ingredients (Ren et al., 2018; Zhang et al., 2018; Fu et al., 2020). External preparation is a vital part of Tibetan medicine, which can avoid first-pass metabolism, deliver less side effects, and be noninvasive compared to other therapies. Baimai ointment (BMO) is a representative formula of Tibetan medicine for external use, which was first recorded in *Clinical Notes of Tibetan Medicine* in the 16th century with a long history, and has its origins in *Four Medical Classics* and other traditional Tibetan medicine classics (Gong et al., 2012; Zhang et al., 2018). Besides, BMO was officially recorded in the Drug Standards of Tibetan Medicine by the Ministry of Health of the People’s Republic of China (Xing-ping et al., 2014). According to the theory of Tibetan medicine, OA belongs to Baimai disease (Ren et al., 2018), and BMO can treat OA with the effect of dispersing cold together with removing dampness, removing blood stasis, and soothing tendons by improving blood circulation (Liu et al., 2007). The effect of BMO in treating OA has been tested in clinical practice for hundreds of years. A randomized clinical trial of 124 OA patients showed that BMO had significant advantages in reducing pain and recovering joint function by employing BMO combined with acupuncture (Wang and Li, 2019). Besides, BMO can be used to alleviate pain after knee replacement surgery (Dingzeng et al., 2020). Studies have shown that the mechanism of BMO in treating OA is related to inflammation and regulation of chondrocyte proliferation and apoptosis (Zhang et al., 2012; Jiang et al., 2019). However, the specific molecular mechanism is still unclear, which limits its further application. Therefore, an in-depth study has to be carried out to further illuminate the mechanism and give theoretical support for the clinical application of BMO.

Network pharmacology is a burgeoning research method for new drug design and development based on the theoretical progress of multidirectional pharmacology, systems biology, and omics (Li et al., 2019). The chemical compositions and active targets of Tibetan medicine are complex, which is consistent with the integrity of network pharmacology (Pang et al., 2018). The molecular docking technology refers to the simulated binding of ligands and receptor proteins *via* a computer platform (Ferreira et al., 2015). In this study, molecular docking was applied to test the binding ability of the key components with key targets. Furthermore, experimental pharmacological studies were employed to reveal the pharmacodynamic effects and mechanisms of BMO against OA.

The research thoroughly illustrated the molecular mechanism of BMO against OA, which can promote the application and development of BMO and may result in more effective therapeutics for OA. Also, this article provided a comprehensive strategy to study the molecular mechanisms of external preparation and gave a new idea for the study of Tibetan medicine.

## Materials and Methods

### Reagents and Materials

Baimai Ointment® was purchased from Qizheng Tibetan Medicine Corporation (Tibet, China). Diclofenac diethylamine Emulgel (Votalin®) was purchased from Novartis Pharma Corporation (Beijing, China). The hematoxylin–eosin (HE) staining kit (C0105) was purchased from Beyotime Biotechnology (Shanghai, China). A toluidine blue staining kit (DA0059) was purchased from Leigen Biotechnology (Beijing, China). The enzyme-linked immunosorbent assay (ELISA) kits including CXCL8, IL-1β, IL-6, MAPK1, tumor necrosis factor (TNF), and VEGFA were obtained from Meimian Biotechnology (Jiangsu, China). All other chemicals used in this study were of analytical grade.

### Collection of Bioactive Compounds and Related Targets

Firstly, the components of *Curcumae longae Rhizoma, Zanthoxyli Pericarpium, Moschus,* and *Glycyrrhizae Radix et Rhizoma*, the main ingredients of BMO, were collected from the traditional Chinese medicines for systems pharmacology database and analysis platform (TCMSP) (http://tcmspw.com/) and traditional Chinese medicine integrated database (TCMID) (http://www.megabionet.org/tcmid/) (Wang et al., 2019; Cui et al., 2020). Drug likeness (DL) ≥ 0.18 was set as the threshold to screen the bioactive components (Wang et al., 2015; Zeng et al., 2021). Since there are too many components in *Glycyrrhizae Radix et Rhizoma*, to facilitate the study, the absorption, distribution, metabolism, and excretion (ADME) parameters were added as follows: half-life (HL) ≥ 4 h (Yang et al., 2014), hydrogen bond donor number (HDON) < 5, and hydrogen bond acceptor number (HACC) < 5 (Lipinski et al., 2001). Secondly, the targets related to the obtained components were screened through the TCMSP database and GeneCards database (https://www.genecards.org/) (Chen and Sun, 2021), and the targets of the components that could not be retrieved in these databases were predicted by the TargetNet database (http://targetnet.scbdd.com/) with an area under the curve ≥7 and prediction probability = 1. Finally, the UniProt database (https://www.uniprot.org/) was used to correct the targets obtained to official names with selected species “*Homo sapiens.*”

### Collection of Osteoarthritis Disease Targets

The targets associated with OA disease were collected through the TCMSP database, GeneCards database, and DisGeNET database (http://www.disgenet.org/web/DisGeNET/menu/home/). After removing the repeated targets, they were corrected to official names with selected species “*Homo sapiens*” using the UniProt database.

### Component–Target Visualization Network Construction

The network of components and related targets of BMO was constructed for visual analysis through Cytoscape 3.6.1 (https://cytoscape.org/). In the network, the nodes with diverse colors and shapes were symbolled for different components and targets. According to the degree of nodes, the key compounds were screened *via* the C-T network.

### Screening of Candidate Targets and Construction of the Protein–Protein Interaction Network

The overlapped genes related to BMO and OA disease were screened as candidate targets using Venny (http://bioinfogp.cnb.csic.es/tools/venny/). The candidate targets were inputted to STRING 11.0 (https://string-db.org/) which includes a great number of protein–protein interaction (PPI) relationships (von Mering et al., 2005). With the confidence score > 0.7 as the screening condition, a PPI network was analyzed after removing the scattered targets. Then, the network information was input into Cytoscape 3.6.1 to visualize the PPI network.

### Gene Ontology and Kyoto Encyclopedia of Genes and Genomes Pathway Enrichment

Gene Ontology (GO) functional and Kyoto Encyclopedia of Genes and Genomes (KEGG) pathway enrichment analyses were conducted with the overlapped targets using the STRING database. According to the biological process (BP), cell composition (CC), molecular function (MF), and the actual roles of the pathways with *p* value < 0.05, we speculated and analyzed the main molecular biological processes or signal pathways of BMO in the treatment of OA. Then, a bubble chart was drawn online using imageGP (http://www.ehbio.com/ImageGP/).

### Molecular Docking

The key components and key targets were obtained from the component–target (C-T) network and PPI network, respectively, for molecular docking. Firstly, the three-dimensional (3D) molecular structures of the core components and chemical drugs used for treating OA were downloaded from the PubChem database (https://pubchem.ncbi.nlm.nih.gov/) in the sdf format, then Chem3D software (https://www.chemdraw.com.cn/) was used to convert them to pdb formats. The 3D structures of core target proteins were downloaded from the Protein Data Bank database (http://www.rcsb.org/) in pdb formats. Secondly, ligands and proteins were prepared through PyMOL (https://pymol.org/) involving charge addition, atomic type assignment, water removing, and extraction of original ligands and ions. Then, the ligands and proteins were saved in the pdbqt format using AutoDock Tools 1.5.6 (http://autodock.scripps.edu/).Thirdly, AutoDock Tools was used for docking after setting grid box parameters. For each ligand, the lowest binding energy was selected as the result of molecular docking. Lastly, the docking conformation analysis and mapping were carried out by PyMOL software, and PLIP (https://projects.biotec.tu-dresden.de/plip-web/plip) was used to analyze the force between ligands and proteins (Yan and Zhou, 2021).

### Establishment of Rat Osteoarthritis Model and Drug Administration

The Sprague Dawley (SD) rats used here were obtained from Vital River Laboratory Animal Technology Co., Ltd., (Beijing, China). All animal experimental procedures were approved by the Animal Ethics Committee of Beijing University of Chinese Medicine (BUCM-4-2019101701-4001). 24 SD rats were randomly divided into four groups, namely, sham-operated group, model group, positive control group, and BMO group. We used a modified Hulth method to establish the rat OA model (Zhou et al., 2018). Firstly, the animals were denied food and water for 8 h before the operation and were fixed in the supine position on the operating table after being anesthetized with ketamine (100 mg/kg) and xylazine (10 mg/kg) by intraperitoneal injection, and applied a tourniquet to the proximal right leg. Secondly, the rats were shaved, and a 4-cm incision was made in the median knee. Then, the medial collateral ligament and medial meniscus of the rats' right knee were cut in all groups except in the sham-operated group, and in sham-operated group, we only opened the joint cavity without destroying any joint structure. Next, the articular cavity and incision were flushed with physiological saline and sutured using a 3–0 absorbable suture. The contralateral knee was placed in the extension position with a plaster after the operation. Penicillin was intramuscularly injected with a dose of 20 U per day to prevent infection within 3 days after the operation.

After operation, BMO and Votalin Emulgel were applied to the right knee joint of the rats in the BMO group and positive control group, respectively, followed by massage for 5 min. The dosage of the ointment was 1 g per time, twice a day. The period of administration was 4 weeks.

### Hematoxylin–Eosin and Toluidine Blue Staining

After 4-week administration, the right knees of all rats were cut and put into 4% paraformaldehyde for fixation, then routinely decalcified with ethylenediaminetetraacetic acid. After being washed, the knee joints were dehydrated by step gradient ethanol and hyalinized by dimethylbenzene. Next, the joints were paraffin-embedded and sliced into 5-μm sections. Neutral gum sealing was performed after conventional HE and toluidine blue staining, and light microscopy used to observe the slices. The modified Mankin score (Table 1) was used to evaluate the cartilage destruction (Mankin et al., 1981).

**TABLE 1:** Modified Mankin scoring criteria.

Organization structure	Microscopic features	Scores
Cartilage structure	Normal	0
	The surface is slightly worn, regular arrangement	1
	Hierarchy disorder, irregular arrangement	2
	The surface is seriously worn, irregular arrangement	3
Toluidine blue staining	Normal	0
	Mild decrease	1
	Moderate decrease	2
	Severe decrease	3
Tide line	Structure of tidal lines is intact	0
	Multilevel structure	1
	Tidal blur	2
	There are blood vessels through the tidal line	3
Proportion of hyaline cartilage and calcified cartilage	Normal	0
	Mild decrease	1
	Moderate decrease	2
	Severe decrease	3

### Enzyme-Linked Immunosorbent Assay

After 4-week administration, the rats in all groups were anesthetized and fixed, and their skin and subcutaneous tissues cut to expose the joint capsule. Then, 400 μl sterile phosphate-buffered saline was injected into the joint cavity. After repeatedly moving the joint, 300 μl joint fluid was carefully extracted for ELISA, which was diluted and added according to the ELISA kit operating instructions. Firstly, it was incubated at 37°C for 30 min, and the concentrated solution was washed thoroughly after dilution. It was then treated to enzymes, warm bath, washing, and color rendering and the termination liquid added to terminate the reaction. CXCL8, IL-1β, IL-6, MAPK1, TNF, and VEGFA were measured within 15 min after the addition of the stop solution. Absorbance was measured immediately at 450 nm using an enzyme marker (Biotech, America), and a standard curve was derived to read the contents of IL-1β, TNF, MAPK1, IL-6, CXCL8, and VEGFA in the joint fluid in different groups.

### Statistical Analysis

Data were presented as the mean ± standard deviation with at least six independent experiments. SPSS 20.0 software was used to perform statistical analyses by one-way analysis of variance, and *p* < 0.05 was considered statistically significant.

## Results

### Active Components of Baimai Ointment

A collection of 44 potential compounds were collected from TCMSP and TCMID databases according to certain criteria, including 7 in *Curcumae longae Rhizoma*, 8 in *Zanthoxyli Pericarpium*, 7 in *Moschus*, and 22 in *Glycyrrhizae Radix et Rhizoma* (Table 2).

**TABLE 2:** Active compounds of BMO. **A.** Active compounds of *Curcumae longae Rhizoma*, *Zanthoxyli Pericarpium*, and *Moschus*.

Herb name	Mol ID	Molecule name	MW	DL
* Curcumae longae Rhizoma*	MOL000449	Stigmasterol	412.77	0.76
	MOL000493	Campesterol	400.76	0.71
	MOL000953	CLR	386.73	0.68
	MOL000892	TNP00001	368.41	0.41
	MOL000951	Dihydrocurcumin	370.43	0.41
	MOL000946	Demethoxycurcumin	338.38	0.33
	MOL000945	Bisdemethoxycurcumin	308.35	0.26
* Zanthoxyli Pericarpium*	MOL013271	Kokusaginin	259.28	0.20
	MOL002663	Skimmianin	259.28	0.20
	MOL002881	Diosmetin	300.28	0.27
	MOL000358	beta-Sitosterol	414.79	0.75
	MOL004368	Hyperin	464.41	0.77
	MOL005093	Diosmin	608.60	0.66
	MOL000514	Nonacosane	408.89	0.39
	MOL000098	Quercetin	302.25	0.28
* Moschus*	MOL010919	17-beta-Estradiol	272.42	0.32
	MOL002359	6-Hydroxy-musizin-8-O-beta-D-glucoside	394.41	0.50
	MOL000987	Cholesterol	384.76	0.67
	MOL002442	Cholesteryl ferulate	562.91	0.63
	MOL000737	Morin	302.25	0.27
	MOL007216	n-Nornuciferine	281.38	0.36
	MOL001232	TES	288.47	0.35

B. Active compounds of *Glycyrrhizae Radix et Rhizoma*.				
Herb name	Mol ID	Molecule name	MW	DL
* Glycyrrhizae Radix et Rhizoma*	MOL001792	DFV	256.27	0.18
	MOL000211	Mairin	456.78	0.78
	MOL002565	Medicarpin	270.30	0.34
	MOL000359	Sitosterol	414.79	0.75
	MOL003896	7-Methoxy-2-methyl isoflavone	266.31	0.20
	MOL000392	Formononetin	268.28	0.21
	MOL004805	Shinflavanone	390.51	0.72
	MOL004806	Euchrenone	406.56	0.57
	MOL004815	Kanzonol B	322.38	0.35
	MOL004835	Glypallichalcone	284.33	0.19
	MOL004838	Kanzonol U	308.35	0.38
	MOL004891	Shinpterocarpin	322.38	0.73
	MOL004910	Glabranin	324.40	0.31
	MOL004941	N1793	256.27	0.18
	MOL004945	Isobavachin	324.40	0.32
	MOL004957	HMO	268.28	0.21
	MOL000497	Licochalcone A	338.43	0.29
	MOL004985	icos-5-enoic acid	310.58	0.20
	MOL004991	7-acetoxy-2-methylisoflavone	294.32	0.26
	MOL004996	Gadelaidic acid	310.58	0.20
	MOL005003	Licoagrocarpin	338.43	0.58
	MOL005018	Xambioona	388.49	0.87

### Component–Target Network Analysis

**FIGURE 1:**
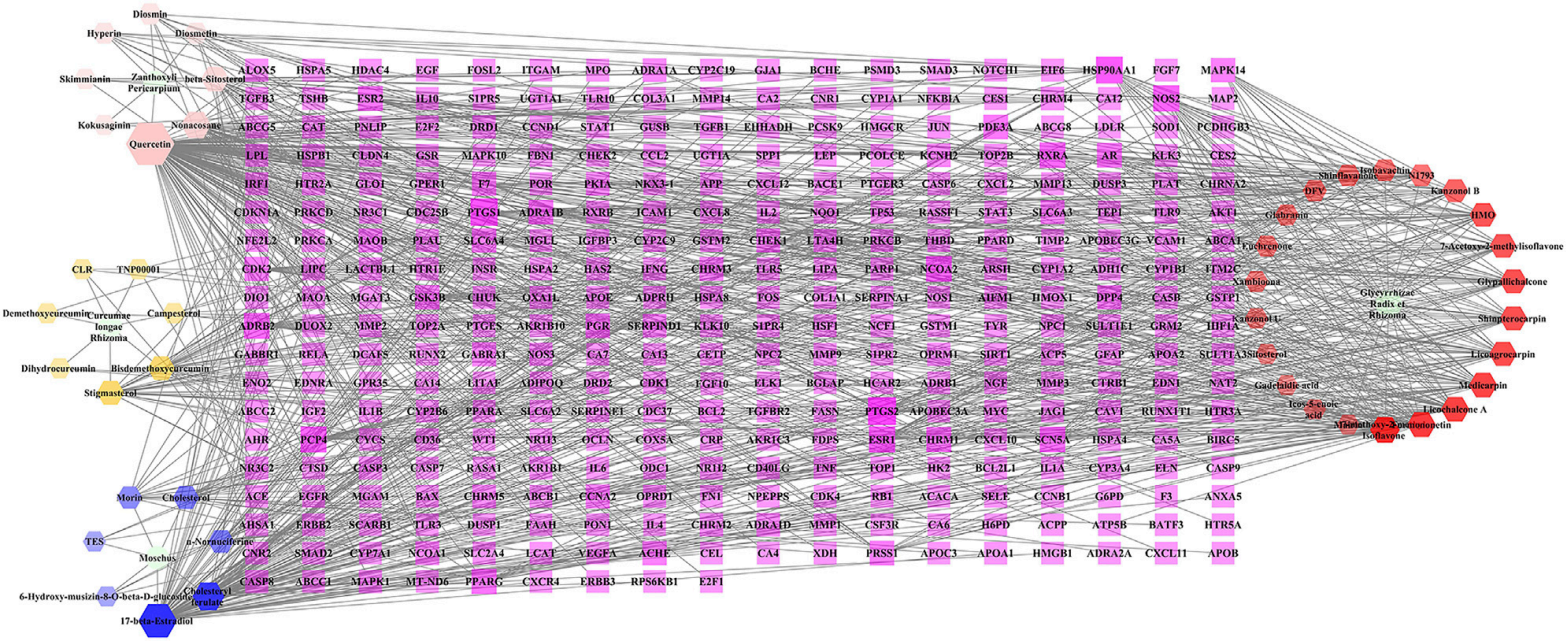
C-T network of BMO. The purple rectangles represented 333 potential target genes of compounds. The hexagons in yellow, pink, blue, and red stand for compounds in *Curcumae longae Rhizoma*, *Zanthoxyli Pericarpium*, *Moschus*, and *Glycyrrhizae Radix et Rhizoma*, respectively. The green ellipse stands for *Curcumae longae Rhizoma*, *Zanthoxyli Pericarpium*, *Moschus*, and *Glycyrrhizae Radix et Rhizoma*. The size and transparency of the nodes were proportional to the degree.

A total of 874 targets corresponding to compounds in BMO were collected from TCMSP, GeneCards, and TargetNet databases, and the remaining 333 targets collected after duplicate removal. The C-T network constructed with Cytoscape 3.6.1 included 381 nodes and 917 edges (Figure 1). The purple rectangles represent the 333 corresponding targets of compounds. The hexagons in yellow, pink, blue, and red stand for compounds in *Curcumae longae Rhizoma*, *Zanthoxyli Pericarpium*, *Moschus*, and *Glycyrrhizae Radix et Rhizoma*, respectively. The green ellipse stands for *Curcumae longae Rhizoma*, *Zanthoxyli Pericarpium*, *Moschus*, and *Glycyrrhizae Radix et Rhizoma*. The size and transparency of the nodes were proportional to the degree. In the network, each compound interacted with 19.86 targets on average, and each target interacted with 2.62 compounds on average. Therefore, the phenomenon that one compound corresponds to multiple targets and one target corresponds to multiple compounds simultaneously exist, which reflects that the traditional Tibetan medicine treats diseases through the mechanism of multicomponent and multi-target. Furthermore, higher degrees indicate more essential nodes in the network. Quercetin, 17-beta-estradiol, and licochalcone A indicated high degrees, and thus might play chief roles in the effect of BMO treating OA. In our previous study, we found that curcumin and glycyrrhizic acid were also involved in the treatment of OA in BMO (Liang et al., 2019).

### Overlapped Targets and Protein–Protein Interaction Network Analysis

**FIGURE 2:**
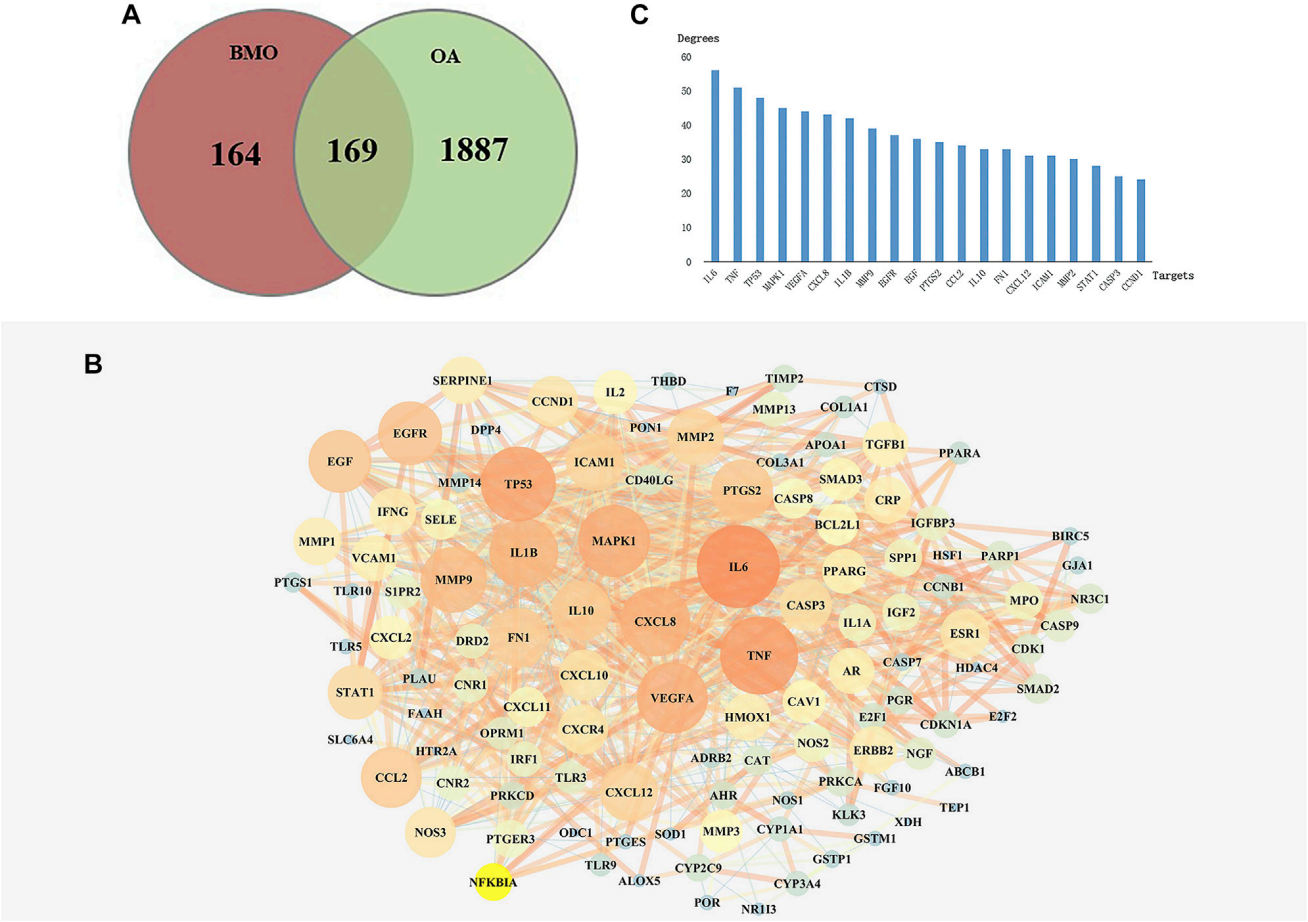
Analysis of key targets of BMO in treating OA. **(A)** After obtaining 333 targets of BMO and 2056 targets of OA, we overlapped the two parts of targets and got 169 mutual targets. **(B)** PPI network of the 169 overlapped targets was analyzed and constructed by STRING and Cytoscape. Larger and red nodes indicate higher degrees. **(C)** Top 20 targets with highest degrees of BMO against OA.

After deleting repeated values and transferring to standard gene symbols, 2056 targets associated with OA were obtained from TCMSP, GeneCards, and DisGeNET databases. Among the 333 compounds and 2054 OA-related targets, there were 169 overlapped targets which were considered potential targets for subsequent studies (Figure 2A). The 169 related targets were input to STRING 11.0 and Cytoscape to analyze and construct a PPI network after removing scattered targets. As shown in Figure 2B, the PPI network included 162 nodes. Larger and red nodes indicated higher degrees. The nodes with the top 20 degrees are shown in Figure 2C, among which the most related targets with high degrees are IL6, TNF, MAPK1, VEGFA, CXCL8, and IL1B. These targets might play a crucial role in the PPI network in treating OA.

### Gene Ontology Analysis

STRING 11.0 was used to analyze the BP, CC, and MF annotations of the 169 potential targets. There were 2082 GO terms (*p* < 0.05), which included 1804 of BP, 183 of MF, and 95 of CC. Most of the BP terms were related to response to inflammatory, regulation of cytokines, cell proliferation, apoptosis, etc., and the main terms of MF were associated with protein binding, receptor binding, cytokine binding, etc. CC enrichment was mainly involved in the cytoplasm, endoplasmic reticulum, organelles, etc.

**FIGURE 3:**
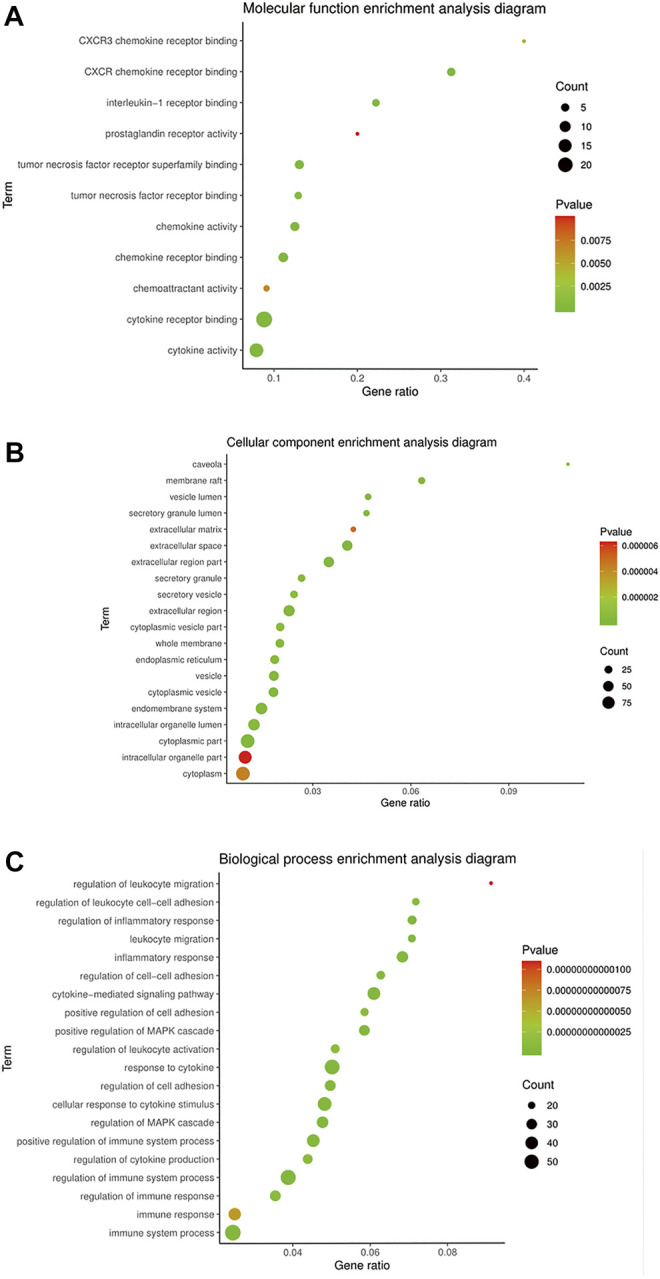
GO enrichment of BMO in the treatment of OA. **(A)** 11 of 183 MF terms related to the mechanism of OA. **(B,C)** Top 20 of CC and BP terms related to the mechanism of OA ranked according to the *p*-value.

The GO terms related to OA could be classified into anti-inflammatory, immunomodulatory, cellular regulation, and metabolism aspects, including 238 of BP and 11 of MF. There were inflammatory response, leukocyte migration, regulation of leukocyte, cell–cell adhesion, regulation of leukocyte activation, interleukin-1 receptor binding, TNF receptor binding, etc. The top 20 terms of BP and CC ranked according to the *p*-value, and 11 terms of MF are shown in Figure 3.

### Kyoto Encyclopedia of Genes and Genomes Analysis

The KEGG pathway annotation indicated that 159 pathways (*p* < 0.05) were enriched, mainly covering diseases, inflammation, immunity, metabolism, and cell regulation. Ranked according to the *p*-value, the top five pathways were pathways in cancer (*p* = 5.42E^−32^), AGE-RAGE signaling pathway in diabetic complications (*p* = 2.65E^−29^), TNF signaling pathway (*p* = 5.85E^−18^), IL-17 signaling pathway (*p* = 1.04E^−17^), and hepatitis B (*p* = 1.04E^−17^).

**FIGURE 4:**
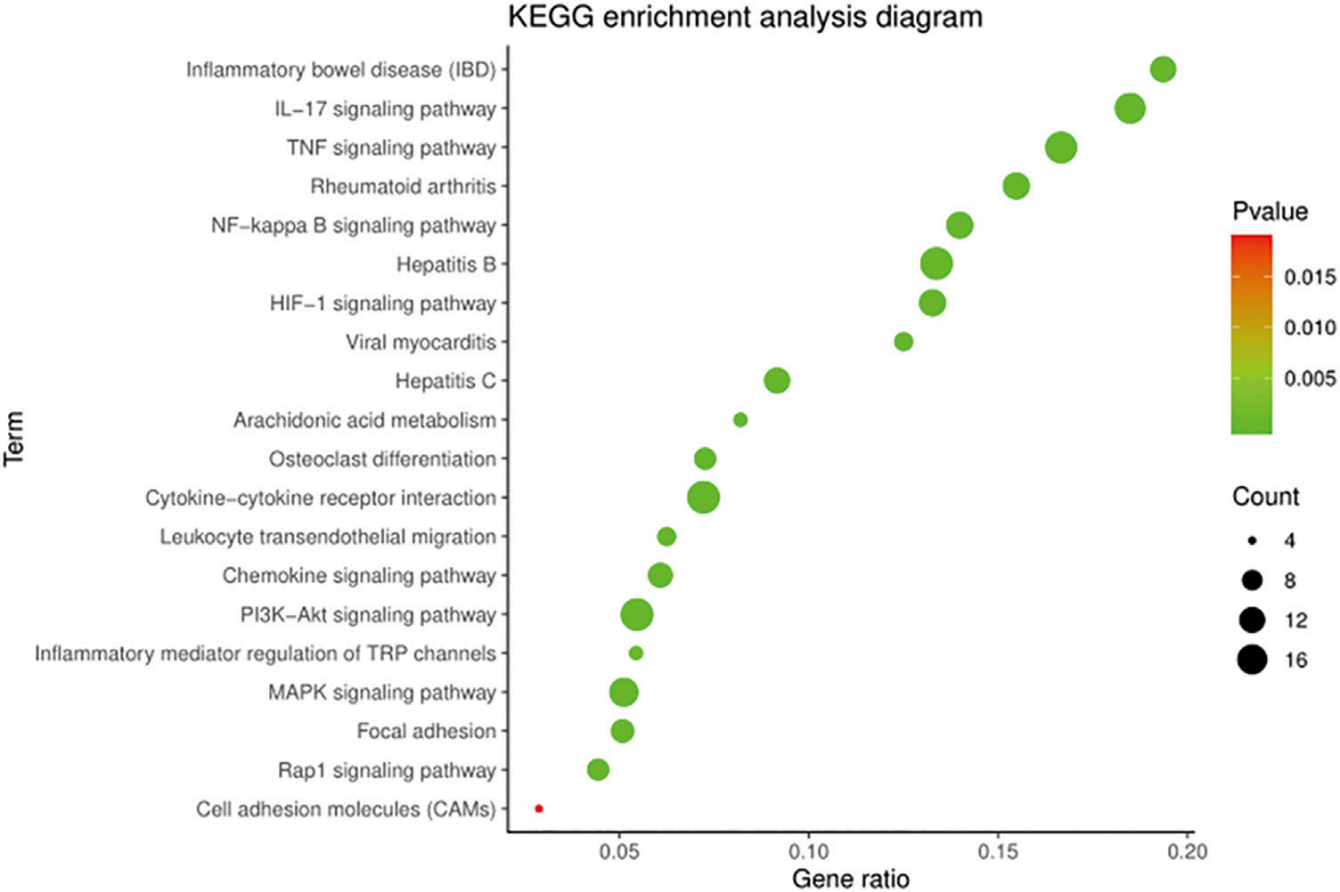
KEGG enrichment of BMO in the treatment of OA. 159 KEGG pathways (*p* < 0.05) were enriched, among which 50 pathways were related to OA. Top 20 of the 50 pathways with the low *p*-value are shown.

On further analysis, there were 50 pathways related to OA, among which 16 pathways were associated with anti-inflammatory, 14 with immunomodulatory, and 20 with cellular metabolism. The KEGG pathways in the top 20 were related to OA with low *p*-values, as shown in Figure 4. The results suggested that the TNF signaling pathway, NF-κB signaling pathway, and HIF-1 signaling pathway played a significant role in treating OA with BMO.

### Molecular Docking

Molecular docking was conducted to verify the binding ability of IL6, TNF, MAPK1, VEGFA, CXCL8, and IL1B with quercetin, 17-beta-estradiol, licochalcone A, curcumin, and glycyrrhizic acid according to network analysis and preliminary research. Meanwhile, chemical drugs used clinically to treat OA such as meloxicam, celecoxib, and nabumetone were also docked with selected target proteins as a reference. The results of the binding energy are shown in Table 3.

**TABLE 3:** Binding energy of key components of BMO with key targets.

Components and chemicals	Binding energy (kcal·mol^−1^)						
	IL6	TNF	MAPK1	VEGFA	CXCL8	IL1B	Average
17-beta-estradiol	−3.75	−4.76	−5.42	−3.66	−4.32	−5.11	−4.50
Curcumin	−1.83	−3.57	−3.52	−2.08	−4.61	−3.25	−3.14
Licochalcone A	−2.28	−3.14	−2.37	−1.58	−2.64	−3.65	−2.61
Quercetin	−1.90	−2.63	−2.27	−2.60	−2.83	−3.31	−2.59
Glycyrrhizic acid	−2.56	−2.87	−2.92	−0.71	−3.08	−2.06	−2.37
Meloxicam	−3.26	−3.91	−3.44	−2.48	−3.86	−4.10	−3.51
Celecoxib	−2.97	−3.57	−3.24	−2.82	−3.75	−3.91	−3.38
Nabumetone	−3.06	−3.86	−2.81	−2.51	−3.69	−3.55	−3.25

The results suggested that the binding energy ranged from −0.71 kcal·mol^−1^ to -5.42 kcal·mol^−1^. Besides, the binding energy was all less than 0 and less than −3.0 kcal·mol^−1^ accounted for 46.67%. When the binding energy was less than 0, it indicated that the ligands could bind to the target proteins freely. The binding energy of key components with key target proteins was equivalent or low compared to that of the chemical drugs, which indicated that the key components of BMO had better binding activity with key targets, thus verifying the reliability of the results of network pharmacology research to a certain extent. The binding energy of 17-β-estradiol with the aforementioned target proteins was even lower than that of the chemical drugs. These components could interact with the key target proteins such as IL6, TNF, MAPK1, VEGFA, CXCL8, and IL1B to have an effect on OA.

**FIGURE 5:**
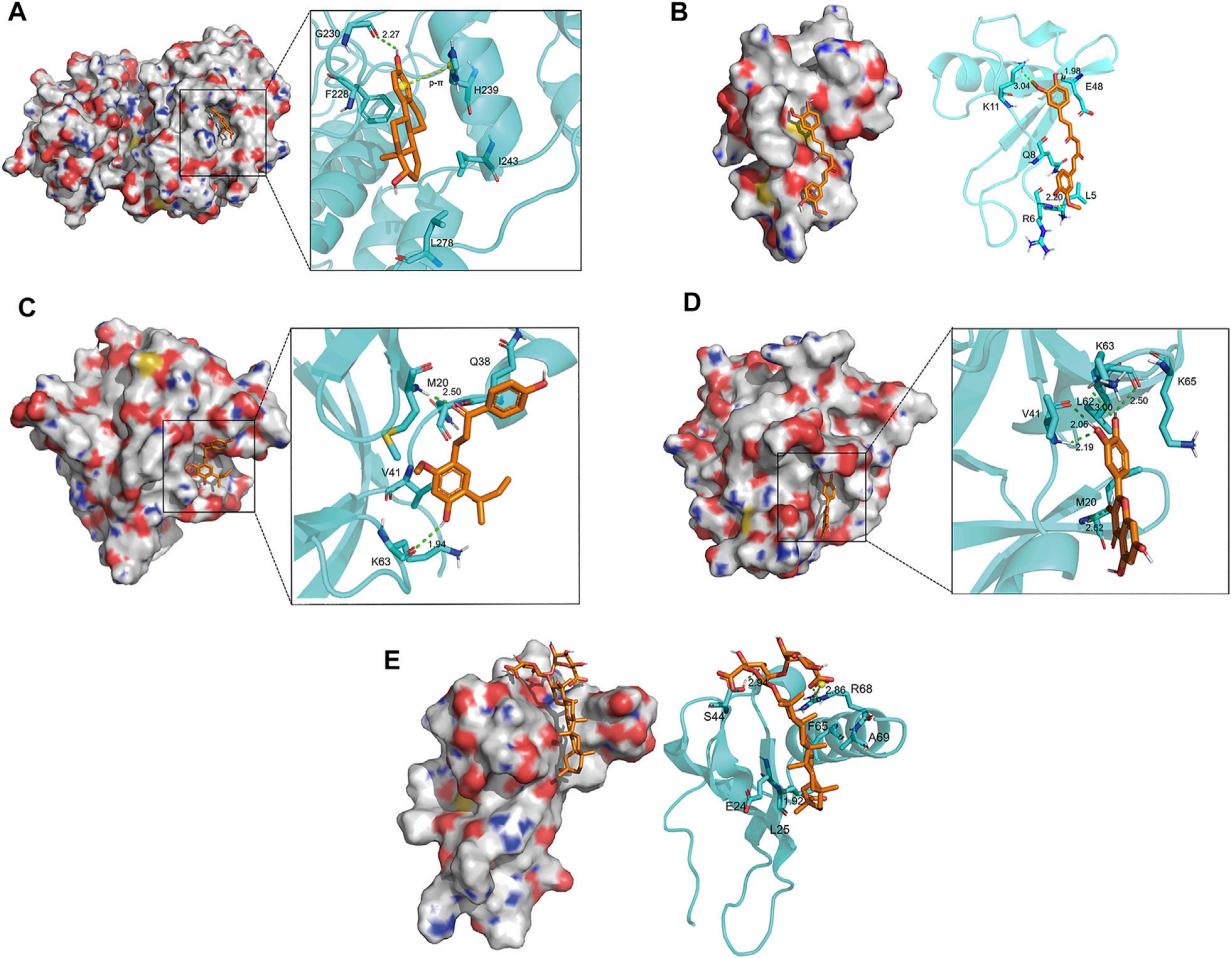
Docking conformation of five key components and their best combined target proteins. **(A)** 17-beta-estradiol bound best to MAPK1. It could form a hydrogen bond with Gly230 in MAPK1 and p-π conjugated with His239. **(B)** Curcumin bound best to CXCL8. It could form three hydrogen bonds with Arg6, Lys11, and Glu48 in CXCL8, respectively. **(C)** Licochalcone A bound best to IL1B. It could form two hydrogen bonds with Met20 and Lys63 in IL1B, respectively. **(D)** Quercetin bound best to ILIB. It could form a hydrogen bond with Met20, two hydrogen bonds with Val41, and two hydrogen bonds with Lys63 in IL1B. **(E)** Glycyrrhizic acid bound best to CXCL8. It could form three hydrogen bonds with Leu25, Ser44, and Arg68 in CXCL8, respectively, and a salt bridge with Arg68.

The docking conformation of the five key components and their best combined target proteins are shown in Figures 5B–F, respectively. As shown in Figure 5A, 17-beta-estradiol could form a structure of hydrogen bond with Gly230 in MAPK1 whose length was 2.27, and the benzene ring in the compound could be p-π conjugated with the pentane ring in His239. As shown in Figure 5B, curcumin could form a structure of three hydrogen bonds with Arg6, Lys11, and Glu48 in CXCL8, and their bond lengths were 2.20, 3.04, and 1.98, respectively. As shown in Figure 5C, licochalcone A could form a structure of two hydrogen bonds with Met20 and Lys63 in IL1B, and their bond lengths were 1.94 and 2.50, respectively. As shown in Figure 5D, quercetin could form a structure of one hydrogen bond with Met20, two hydrogen bonds with Val41, and two hydrogen bonds with Lys63 in IL1B and their bond lengths were 2.62, 2.19, 2.06, 3.00, and 2.50, respectively. As shown in Figure 5E, glycyrrhizic acid could form a structure of three hydrogen bonds with Leu25, Ser44, and Arg68 in CXCL8 and their bond lengths were 1.92, 2.94, and 2.86, respectively. Besides, it could form a salt bridge with Arg68 in CXCL8, which enhanced the stability of binding.

In conclusion, there was a stable interaction between key compounds and key target proteins with different bonds. The results illustrated that the key compounds could bind to the active site of key target proteins to have an effect on treating OA.

### Pathology

**FIGURE 6:**
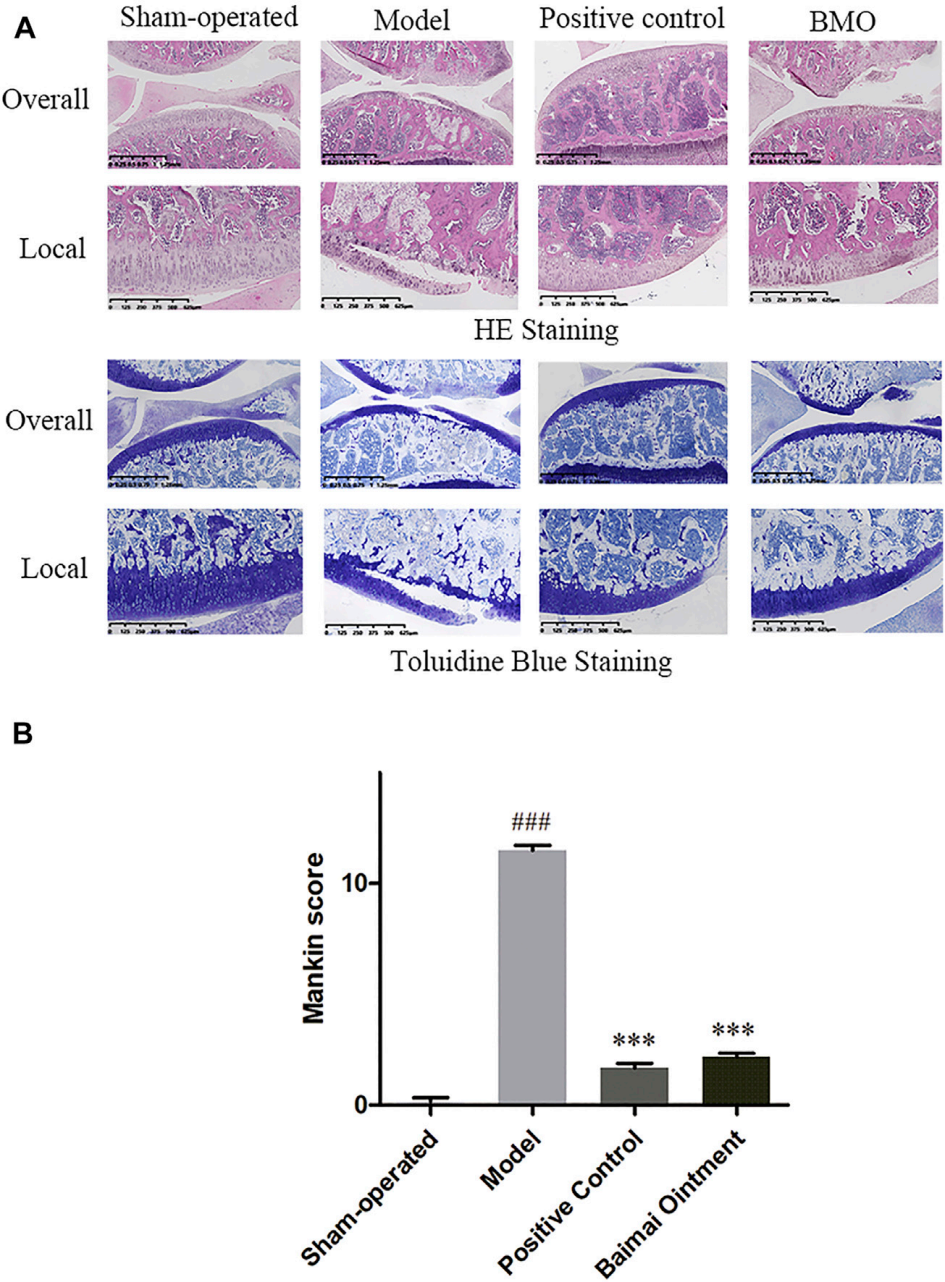
HE and toluidine blue staining of knee joints of SD rats in each group after 4 weeks (*n* = 6). **(A)** In the sham-operated group, the joints were in regular condition, and toluidine blue staining was clear. In the model group, the meniscus was lost, the cartilage surface was seriously worn, and the arrangement of chondrocytes was disordered. Toluidine blue staining of cartilage tissue was seriously depigmented. After administration, the joints were in better condition. **(B)** Comparison of the Mankin score of knee joints of rats in each group. ^###^ *p* < 0.001 vs. Sham-operated, ^***^ *p* < 0.001 vs. Model.

The knee joints of rats in each group were observed by HE and toluidine blue staining (Figure 6A). In the sham-operated group, the meniscus could be seen on the knee joint of rats in the medial side, and the cartilage surface was neat without cartilage wear and rupture. The cartilage was thick, and the chondrocytes were arranged in order. The cartilage of the tibial plateau could be divided into hyaline cartilage above and calcified cartilage below. Toluidine blue staining was clear. In the model group, the meniscus of the knee joint was lost, and the cartilage surface was seriously worn with its layer thinner. The arrangement of chondrocytes was disordered, and the tide line was not clear. The proportion of the hyaline cartilage and calcified cartilage decreased, and toluidine blue staining of the cartilage tissue was seriously depigmented, which indicated that the OA model was successfully established. Compared with the model group, the positive control group and BMO group were all in better conditions. The cartilage surface was slightly worn, and its layer was thicker than the model group. After treating with drugs, the chondrocytes arranged well, and the tidal line was clearly visible. The ratio of the hyaline cartilage to calcified cartilage increased, and toluidine blue staining was clear on the surface of the cartilage tissue. By comparing the modified Mankin score of each group (Figure 6B), we found that there were significant differences (*p* < 0.001) between the sham-operated group and model group. After administration, the scores decreased significantly (*p* < 0.001). There was no significant difference between the positive control group and BMO group (*p* > 0.05).

### Expression of CXCL8, IL-1β, IL-6, MAPK1, TNF, and VEGFA in Joint Fluid

**FIGURE 7:**
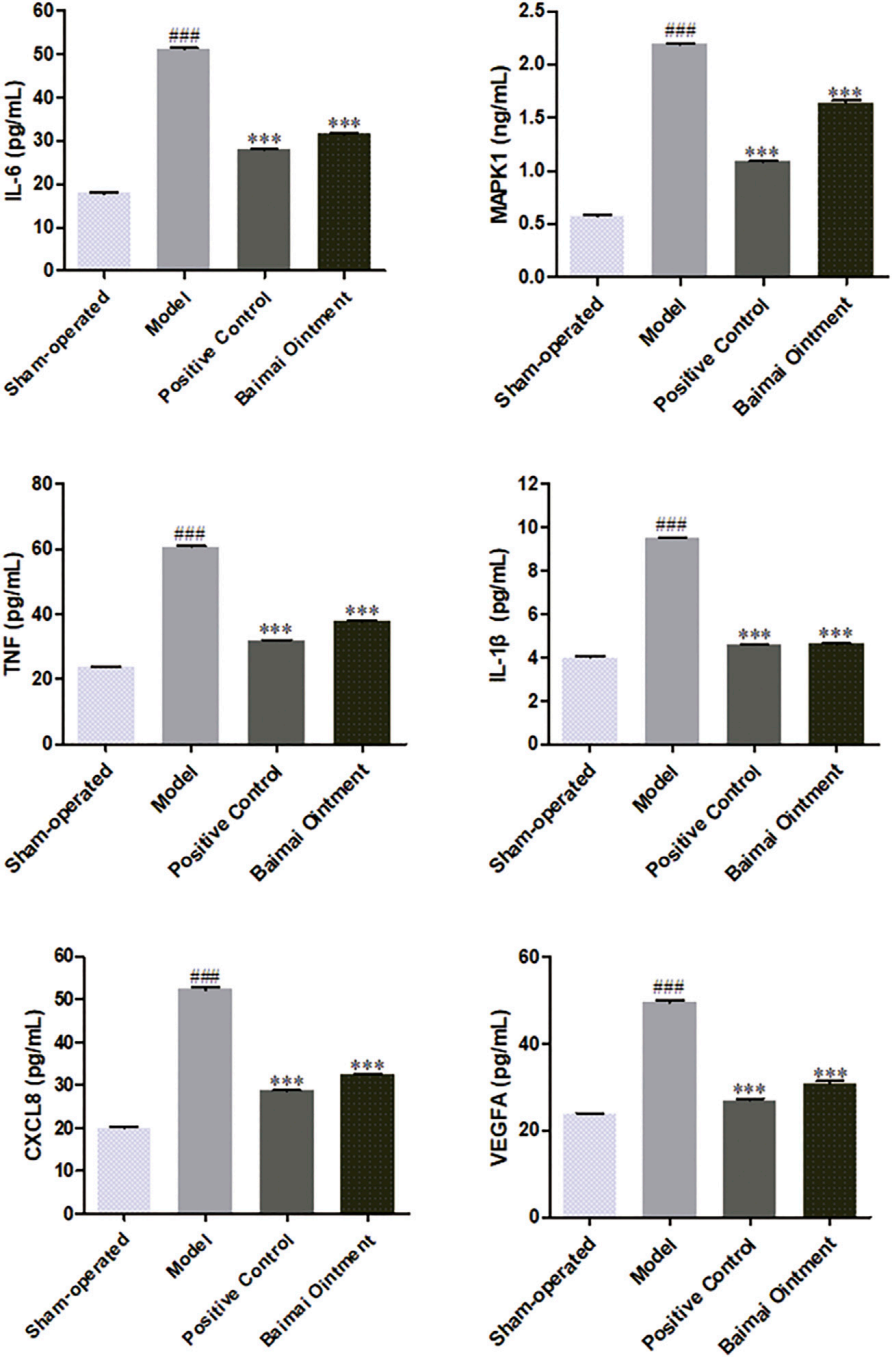
Expression of IL-6, MAPK1, TNF, IL-1, CXCL8, and VEGFA proteins in the joint fluid of rats in each group after 4 weeks (*n* = 6). ^###^ *p* < 0.001 vs. Sham-operated, ^***^ *p* < 0.001 vs. Model.

The levels of CXCL8, IL-1β, IL-6, MAPK1, TNF, and VEGFA in joint fluid were detected by ELISA. As shown in Figure 7, compared to the sham-operated group, the levels of those cytokines both increased significantly (*p* < 0.001) in the model group, indicating the successful establishment of the OA model. After administration, the levels of those cytokines decreased significantly (*p* < 0.001) in both the BMO group and positive control group, which suggested BMO had effects on treating OA to a certain extent by regulating CXCL8, IL-1β, IL-6, MAPK1, TNF, and VEGFA in the joint fluid.

## Discussion

Among all kinds of arthritis, OA is the most common one, which is called degenerative joint disease or *wear and tear* arthritis as well. The lesions of the disease that occur most frequently are in the knees, hips, and hands. OA is usually treated with combined therapies including medications, surgery, and exercise (Fortin et al., 2002; Totlis et al., 2021). Medication therapy is of most importance in treating OA, which includes nonsteroidal anti-inflammatory drugs, cartilage protectants, and intra-articular injection drugs in Western medicine (Lane and Thompson, 1997; Petrella and Wakeford, 2015). Compared to Western medicine that treats with a single target, traditional medicine such as traditional Tibetan medicine has its unique regulation effects with multi-target and multi-pathway, which overcome the shortages of drug resistance and unilateral effects (Liu et al., 2019; Yu et al., 2020).

BMO is a famous formula for external use in treating OA, which consists of 11 traditional Tibetan medicines such as *Curcumae longae Rhizoma*, *Zanthoxyli Pericarpium*, *Moschus*, *Glycyrrhizae Radix et Rhizoma*, *Myristicae Semen*, *Kaempferia galanga Linn*, and so on (Gong et al., 2012; Xing-ping et al., 2014)*.* According to the traditional theory of Tibetan medicine and reported research, *Curcumae longae Rhizoma*, *Zanthoxyli Pericarpium*, *Moschus*, and *Glycyrrhizae Radix et Rhizoma* were considered the main ingredients of BMO against OA with their anti-inflammation and other pharmacological actions. *Curcumae longae Rhizoma* is the sovereign medicine of BMO, which has the effect of blood-breaking and promoting Qi, dredging channels, and relieving pain. Curcumin is the major and effective component of *Curcumae longae Rhizoma*, which is applied as a nonsteroidal anti-inflammatory drug in the clinic and has a significant effect on OA (Buhrmann et al., 2011). *Zanthoxyli Pericarpium* and *Moschus* belong to warm–hot natured drugs, which possess the functions of promoting blood circulation and relieving pain (Wang et al., 2014; Kim et al., 2019). Besides, *Glycyrrhizae Radix et Rhizoma* has been widely reported to have anti-inflammatory and antioxidant effects, etc. (Feng et al., 2013; Tavvafian et al., 2020).

However, the molecular mechanism of BMO has not been clarified. In this study, we adopted a network pharmacology approach together with molecular docking and experimental validation to investigate the bioactive compounds and molecular mechanisms of BMO in treating OA. From the network pharmacology studies, bioactive compounds and gene targets of BMO were obtained, and 169 intersection targets of BMO and OA were screened. Combined with C-T network analysis and previous studies (Liang et al., 2019), glycyrrhizic acid, 17-β-estradiol, curcumin, licochalone A, and quercetin were considered the candidate key components, and IL6, TNF, MAPK1, VEGFA, CXCL8, and IL1B were the candidate key targets in treating OA. The KEGG indicated that the TNF signaling pathway, NF-κB signaling pathway, and HIF-1 signaling pathway were the potential pathways. Molecular docking implied a strong combination between key components and key targets. The pathology and animal experiments showed BMO had a great effect on OA *via* regulating IL6, TNF, MAPK1, VEGFA, CXCL8, and IL1B targets. These findings are consistent with the results obtained from the network pharmacology approach.

Through network pharmacology research, a total of 44 potential compounds were screened according to the ADME parameters such as DL, HL, HDON, and HACC. To further analyze, a C-T network was constructed to show the interaction between compounds and targets, among which 17-β-estradiol, licochalcone A, and quercetin showed higher degrees. The higher the degree value, the more targets the compounds will interact with. Our previous study has shown that glycyrrhizic acid and curcumin were the major effective compounds of BMO which could enter into the blood circulation *via* transdermal delivery. Thus, glycyrrhizic acid, curcumin, 17-β-estradiol, licochalcone A, and quercetin were determined as the candidate key compounds of BMO. Studies have shown that the above compounds have significant anti-inflammatory effects and can act on related cytokines such as IL-1 β, TNF α, and other targets, and the NF-κB pathway plays a role in treating OA. Researches have shown that glycyrrhizic acid does well in anti-inflammatory and immunomodulatory effects. Naemehsadat T et al. demonstrated that glycyrrhizic acid could significantly decrease serum TNF-α, IL-6, and IL-1β in overweight young men to decrease muscular damage (Tavvafian et al., 2020). Feng et al. (2013) found that glycyrrhizic acid could cause the inhibition of the RAGE/NF-κB pathway, thus having anti-apoptotic, anti-inflammatory, and antioxidative stress effects, which was demonstrated to have a protective effect on AGEs-induced endothelial dysfunction. 17-β-estradiol is the major component of estrogen, which was considered to have an association with developing knee OA in middle-aged women (Sowers et al., 2006). Huang et al. (2011) established an OA model in rats and cultured chondrocytes to investigate the function of 17-β-estradiol, which showed that 17-β-estradiol could regulate the PI3K/Akt pathway to promote cell proliferation in chondrocytes. It has been widely reported that curcumin has a significant therapeutic effect on OA by regulating cytokines such as IL6, TNF-α, IL1B, and TNF, and signaling pathways such as IL-17 and NF-kB signaling pathways (Sylvester et al., 2004; Zhang and Zeng, 2019). Licochalcone A has a significant anti-inflammatory effect *via* regulating various pathways such as MAPK, NF-κB, and Wnt/beta-catenin signaling pathways (Chen et al., 2017; Lien et al., 2017). Yang et al. (2018) revealed that licochalcone A inhibited the progression and development of arthritis *in vivo via* suppressing NF-κB activation. Jia et al. (2017) demonstrated that licochalcone A could activate the Nrf2 signaling pathway to show anti-inflammatory effects in rat chondrocytes stimulated by IL1β. Quercetin has been reported to have effects on various OA animal models. Hu et al. (2019) revealed that quercetin had a great alleviation effect on the rat OA model *via* inhibiting inflammation and apoptosis of chondrocytes, having synovial macrophages polarized to M2 macrophages. Qiu et al. (2018) verified that quercetin upregulated the AMPK/SIRT1 signaling pathway to attenuate mitochondrial dysfunction and biogenesis in OA rats.

To obtain the potential targets of BMO in treating OA, we intersected the targets of BMO and OA and got a total of 169 overlapped targets. The PPI network was constructed to analyze the relationship between these overlapped target proteins. Moreover, the higher degree value represents the more important part the target takes in. The targets with high degrees were IL6, TNF, MAPK1, VEGFA, CXCL8, and IL1B, and they were determined as key targets. Next, molecular docking has been conducted, and the binding energy of key compounds and key target proteins was low which indicated strong affinities between them. According to the results of animal experiments, BMO had a great therapeutic effect on OA in rats, and its mechanism for treating OA was mainly through regulating the key target proteins mentioned above. It has been reported that these targets are involved in inflammation, immunity, and apoptosis, which greatly contribute to the pathogenesis of OA. IL6, derived from macrophages, chondrocytes, and osteoclasts can increase inflammatory cells in synovial tissue and stimulate chondrocyte proliferation. Dewa GK et al. found that the incidence of lumbar OA in patients with high levels of IL6 was five times higher than in patients with low levels of IL6, suggesting that IL6 is closely related to the occurrence of OA (Pratama et al., 2019). TNF, derived from macrophages and chondrocytes, serves as an indispensable mediator in the degradation of the cartilage matrix and is closely related to synovitis, and it has been widely reported that many drugs interact with TNF to make an effect on OA. Yuan et al. (2010) probed into the mechanism of Juanbi Capsules in guarding against knee OA in the rabbit and found it possible to decrease serum TNF-α, IL-1, and IL-6 contents to prevent OA. MAPK family is a group of evolutionarily conserved serine/threonine kinases, which can regulate inflammation and apoptosis. Studies have shown that BMO can modify the expression of MAPK (Guo et al., 2009). The increased level of VEGFA is related to the progression of OA. It is involved in the special pathological process of OA, including cartilage degeneration, osteophyte formation, subchondral bone cysts and sclerosis, synovitis, and pain (Nelson, 2017). Inhibition of VEGFA signal transduction can reduce the progression of OA (Hamilton et al., 2016). Research has shown that IL1β expression could increase by advanced oxidation protein products in rat chondrocytes, then the progress of cartilage degeneration accelerated in OA, which illustrated the relationship between IL-1β and OA (Liao et al., 2020). Yang et al. (2016) found that CXCL8 and CXCL11 could accelerate cell apoptosis and suppress chondrocytes proliferation, which is believed to be related to the regulation of the JAK-STAT signaling pathway as well as the NF-κB and MAPK pathways and the enhancement of other proinflammatory cytokines expressions. Therefore, CXCL8/11 may exacerbate the disease progression of OA, and it may be designed as a new therapeutic target for OA.

2082 GO terms and 159 KEGG pathways have been obtained by GO and KEGG enrichment. After analyzing each pathway, it showed that BMO could regulate immune function and inhibit inflammatory reactions through inflammatory, immune, and cellular metabolism pathways, so as to achieve clinical therapeutic effects on OA. The representative pathways include TNF, NF-κB, and HIF-1 pathways. The TNF signaling pathway is mediated by related inflammatory factors such as TNF-α, which mainly play the role of regulating inflammation. There are many genes related to inflammation in the NF-κB pathway, so it has chronic activity in many inflammatory diseases, especially, in OA. The NF-κB pathway is closely associated with the pathophysiology of OA *via* a variety of effects and can be activated in OA chondrocytes in the course of aging and inflammation (Marcu et al., 2010; Saito, 2020). Wu et al. (2020) demonstrated that stachydrine protected chondrocytes from IL-1β–induced inflammation through the the NF-κB pathway. Similarly, the HIF-1 pathway involves a transcription factor in the basic helix–ring–helix–PAS domain, which is very essential in response to hypoxia. Studies have shown that upregulation of HIF-2α contributes to the progression of OA *via* inducing primary cilia loss, also it might be designed as therapeutic targets for OA (Yang et al., 2019). Li et al. (2020) found that knee OA induced by monoiodoacetic acid could be suppressed *via* inhibiting HIF-1α/NLRP3 inflammasome signaling.

The current study exhibits several limitations though. Firstly, the network pharmacology method is based on the existing research results to explore the mechanism of the prescription. There may be a small number of pharmaceutical ingredients and action targets that have not been reported yet, and OA-related targets are also constantly updated, so this study may not be sufficient. In addition, oral bioavailability ≥ 30% and DL ≥ 0.18 were normally used as screening criteria in many studies. In this study, BMO is an external preparation, then the screening criterion was only DL ≥ 0.18, which may omit some active compounds (e.g., the DL of glycyrrhizic acid is 0.11). Lastly, although molecular docking technology has been widely used in various drug research fields, it still has some limitations just as a computer virtual screening method. For example, quercetin and cholesteryl ferulate, the main components of BMO screened in network pharmacology, had high binding energy with targets, which meant difficulty in combining. It may be because the target is not fixed and may change with the environment *in vivo*, which cannot be simulated by molecular docking. In this study, the combination of the two methods to explore the molecular mechanism of BMO in the treatment of OA and experimental validation was necessary to overcome the limitations to a certain extent.

## Conclusion

**FIGURE 8:**
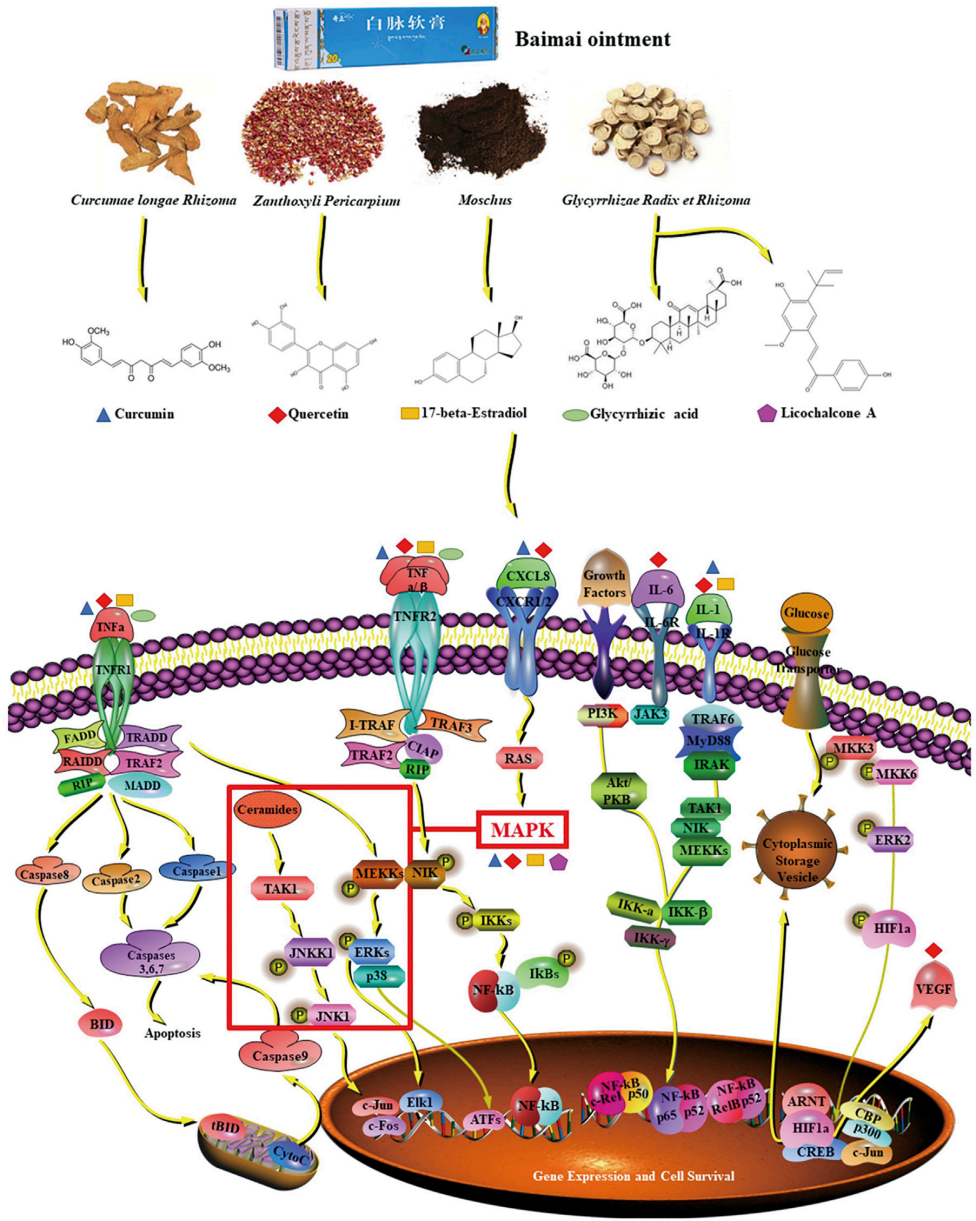
Mechanism of BMO in the treatment of OA. The five key compounds of four main ingredients in BMO could interact with IL-6, MAPK1, TNF, IL-1, CXCL8, and VEGFA, and these key targets were involved in the TNF pathway, NF-κB pathway, and HIF-1 pathway.

In this study, it was found that BMO had multicomponents and multi-targets in treating OA. The possible molecular mechanism of action is that the candidate key components of glycyrrhizic acid, 17-β-estradiol, curcumin, licochalcone A, and quercetin act on IL6, TNF, MAPK1, VEGFA, CXCL8, and IL1B and these key targets regulate the TNF signaling pathway, NF-κB signaling pathway, and HIF-1 signaling pathway, so as to play a therapeutic role (Figure 8). The verification of molecular docking and pharmacological experiments demonstrated that the key components and key target proteins obtained from network pharmacology research combined well. The result of the network pharmacology study is reliability, and it is reasonable to explain the mechanism of BMO in treating OA. The study demonstrated that BMO had a great therapeutic effect on OA in the rat model for the first time, which could partly recover the lesion of joints and significantly decrease cytokines such as CXCL8, IL-1β, IL-6, MAPK1, TNF, and VEGFA to alleviate inflammation.

Traditional medicine is a valuable source for disease treatment, such as Tibetan medicine has abundant applications in the clinic. Hence, to study its mechanism for treatment is necessary. However, its molecular mechanism is difficult to reveal because of complicated components. This study combined network pharmacology and molecular docking methods with pharmacological experiments to uncover the molecular mechanism of Tibetan medicine BMO in treating OA from three aspects of components, targets, and pathways. The strategy we adopted was both reliable and efficient to study the molecular mechanism of external preparation with a combination of theoretical pharmacology and experimental pharmacology.

The significance of this study is preliminarily clarifying the molecular mechanism of BMO in treating OA, thus providing theoretical foundation for clinical application and further research. The obtained candidate key components may be designed for new drugs due to their great effects. Also, the key targets and potential pathways selected can provide new ideas and directions for targeted intervention therapy in treating OA in future development. With the progress of bioinformatic technologies such as molecular fishing, molecular dynamics simulation, and so on, the study of molecular mechanism and drug design has been efficient. Later, binding with advanced bioscience techniques such as DNA chips, single-cell sequence, and surface plasmon resonance can be an effective verification. The theoretical–experimental research pattern may serve as a universal method in drug study.

## Data Availability

The original contributions presented in the study are included in the article/Supplementary Material; further inquiries can be directed to the corresponding authors.

## Abbreviations

ADME, absorption, distribution, metabolism, and excretion; AUC, area under the curve; ANOVA, analysis of variance; BP, biological process; BMO, Baimai ointment; CC, cell composition; C-T, component–target; DL, drug likeness; EDTA, ethylenediaminetetraacetic acid; ELISA, enzyme-linked immunosorbent assay; GO, Gene Ontology; HE, hematoxylin–eosin; HL, half-life; HDON, hydrogen bond donor number; HACC, hydrogen bond acceptor number; KEGG, Kyoto Encyclopedia of Genes and Genomes; MF, molecular function; OA, osteoarthritis; OB, oral bioavailability; PBS, phosphate-buffered saline; PDB, Protein Data Bank; PPI, protein–protein interaction; SD, Sprague Dawley; TCMID, traditional Chinese medicine integrated database; TCMSP, traditional Chinese medicines for systems pharmacology database and analysis platform.
