# Supplementary material for: Study on the Mechanism of Baimai Ointment in the Treatment of Osteoarthritis Based on Network Pharmacology and Molecular Docking with Experimental Verification
Source: Front Genet. 2021 Nov 17;12:750681. doi: 10.3389/fgene.2021.750681 (PMC8635803; doi:10.3389/fgene.2021.750681)
Supplement: Supplementary file 1 [file DataSheet1.pdf]

Table S1. Overlapped targets of Baimai ointment and osteoarthritis

Gene name	Protein name
PGR	Progesterone receptor
PTGS1	Prostaglandin G/H synthase 1
PTGS2	Prostaglandin G/H synthase 2
ADRB2	Beta-2 adrenergic receptor
PLAU	Urokinase-type plasminogen activator
HTR2A	5-hydroxytryptamine 2A receptor
NR3C1	Glucocorticoid receptor
ESR1	Estrogen receptor
TLR3	Toll Like Receptor 3
TLR5	Toll Like Receptor 5
TLR9	Toll-like receptor 9
TLR10	Toll Like Receptor 10
MMP9	Matrix metalloproteinase-9
MMP14	Matrix Metallopeptidase 14
TIMP2	TIMP Metallopeptidase Inhibitor 2
ABCB1	ATP-dependent translocase ABCB1
PPARG	Peroxisome Proliferator Activated Receptor Gamma
CYP3A4	Cytochrome P450 3A4
CYP2C9	Cytochrome P450 Family 2 Subfamily C Member 9
NOS2	Nitric oxide synthase
DPP4	Dipeptidyl peptidase IV
SLC6A4	Sodium-dependent serotonin transporter
OPRM1	Mu-type opioid receptor
CASP9	Caspase-9
CASP3	Caspase-3
CASP8	Caspase-8
PRKCA	Protein kinase C alpha type
TGFB1	Transforming growth factor beta-1
PON1	Serum paraoxonase/arylesterase 1
CNR1	Cannabinoid receptor 1
CNR2	Cannabinoid receptor 2
DRD2	D(2) dopamine receptor
NOS3	Nitric oxide synthase
NOS1	Nitric oxide synthase
HDAC4	Histone deacetylase 4
S1PR2	Sphingosine 1-phosphate receptor 2
FAAH	Fatty-acid amide hydrolase 1
ALOX5	Arachidonate 5-lipoxygenase
AR	Androgen receptor
MMP3	Stromelysin-1
F7	Coagulation factor VII

EGFR	Epidermal growth factor receptor
VEGFA	Vascular endothelial growth factor A
CCND1	G1/S-specific cyclin-D1
BCL2L1	Bcl-2-like protein 1
CDKN1A	Cyclin-dependent kinase inhibitor 1
EIF6	Eukaryotic translation initiation factor 6
MMP2	72 kDa type IV collagenase
MAPK1	Mitogen-activated protein kinase 1
IL10	Interleukin-10
EGF	Pro-epidermal growth factor
TNF	Tumor necrosis factor
IL6	Interleukin-6
TP53	Cellular tumor antigen p53
NFKBIA	NF-kappa-B inhibitor alpha
POR	NADPH--cytochrome P450 reductase
ODC1	Ornithine decarboxylase
XDH	Xanthine dehydrogenase/oxidase
SOD1	Superoxide dismutase [Cu-Zn]
MMP1	Interstitial collagenase
STAT1	Signal transducer and activator of transcription 1-alpha/beta
CDK1	Cyclin-dependent kinase 1
ERBB2	Receptor tyrosine-protein kinase erbB-2
HMOX1	Heme oxygenase 1
CAV1	Caveolin-1
GJA1	Gap junction alpha-1 protein
CYP1A1	Cytochrome P450 1A1
ICAM1	Intercellular adhesion molecule 1
IL1B	Interleukin-1 beta
CCL2	C-C motif chemokine 2
SELE	E-selectin
VCAM1	Vascular cell adhesion protein 1
PTGER3	Prostaglandin E2 receptor EP3 subtype
CXCL8	Interleukin-8
BIRC5	Baculoviral IAP repeat-containing protein 5
IL2	Interleukin-2
CCNB1	G2/mitotic-specific cyclin-B1
THBD	Thrombomodulin
SERPINE1	Plasminogen activator inhibitor 1
COL1A1	Collagen alpha-1(I) chain
IFNG	Interferon gamma
IL1A	Interleukin-1 alpha
MPO	Myeloperoxidase
HAS2	Hyaluronan synthase 2
GSTP1	Glutathione S-transferase P

PARP1	Poly [ADP-ribose] polymerase 1
AHR	Aryl hydrocarbon receptor
COL3A1	Collagen alpha-1(III) chain
CXCL11	C-X-C motif chemokine 11
CXCL2	C-X-C motif chemokine 2
NR1I3	Nuclear receptor subfamily 1 group I member 3
PPARA	Peroxisome proliferator-activated receptor alpha
HSF1	Heat shock factor protein 1
CRP	C-reactive protein
CXCL10	C-X-C motif chemokine 10
SPP1	Osteopontin
E2F1	Transcription factor E2F1
E2F2	Transcription factor E2F2
CTSD	Cathepsin D
IGFBP3	Insulin-like growth factor-binding protein 3
IGF2	Insulin-like growth factor II
CD40LG	CD40 ligand
IRF1	Interferon regulatory factor 1
DIO1	Type I iodothyronine deiodinase
GSTM1	Glutathione S-transferase Mu 1
CA2	Carbonic anhydrase II
FGF10	Fibroblast growth factor 10
CAT	Catalase
TEP1	Telomerase protein component 1
MMP13	Collagenase 3
CASP7	Caspase-7
NGF	Beta-nerve growth factor
PRKCD	Protein kinase C delta type
FN1	Fibronectin
TYR	Tyrosinase
SMAD2	Mothers against decapentaplegic homolog 2
CXCL12	Stromal cell-derived factor 1
CXCR4	C-X-C chemokine receptor type 4
PTGES	Prostaglandin E synthase
KLK3	Prostate-specific antigen
APOA1	Apolipoprotein A-I
SMAD3	Mothers against decapentaplegic homolog 3
TGFB3	Transforming growth factor beta-3
BGLAP	Osteocalcin
LEP	Leptin
ITGAM	Integrin alpha-M
FGF7	Keratinocyte growth factor
TGFBR2	TGF-beta receptor type-2
ELN	Elastin

FBN1	Fibrillin-1
DUSP1	Dual specificity protein phosphatase 1
ACP5	Tartrate-resistant acid phosphatase type 5
ABCA1	ATP Binding Cassette Subfamily A Member 1
APOE	Apolipoprotein E
APOB	Apolipoprotein B
SCARB1	Scavenger Receptor Class B Member 1
LPL	Lipoprotein Lipase
NPC1	NPC Intracellular Cholesterol Transporter 1
HMGCR	3-Hydroxy-3-Methylglutaryl-CoA Reductase
ARSH	Arylsulfatase Family Member H
APP	Amyloid Beta Precursor Protein
PNLIP	Pancreatic Lipase
ACE	Angiotensin I Converting Enzyme
ADPRH	ADP-Ribosylarginine Hydrolase
EDN1	Endothelin-1
RPS6KB1	Ribosomal Protein S6 Kinase B1
ANXA5	Annexin A5
SERPINA1	Serpin Family A Member 1
HMGB1	High Mobility Group Box 1
ENO2	Enolase 2
GFAP	Glial Fibrillary Acidic Protein
GUSB	Glucuronidase Beta
SIRT1	NAD-dependent protein deacetylase sirtuin-1
COX5A	Cytochrome C Oxidase Subunit 5A
G6PD	Glucose-6-Phosphate Dehydrogenase
OXA1L	OXA1L Mitochondrial Inner Membrane Protein
CD36	Platelet glycoprotein 4
GSR	Glutathione reductase, mitochondrial
HTR3A	5-hydroxytryptamine receptor 3A
OPRD1	Delta-type opioid receptor
ESR2	Estrogen receptor beta
MAPK10	Mitogen-activated protein kinase 10
CDK2	Cell division protein kinase 2
CCNA2	Cyclin-A2
MAPK14	Mitogen-activated protein kinase 14
GSK3B	Glycogen synthase kinase-3 beta
IL4	Interleukin-4
STAT3	Signal transducer and activator of transcription 3
CDK4	Cell division protein kinase 4

Table S2. GO enrichment of Baimai ointment in the treatment of osteoarthritis

A. Molecular function enrichment (11 pathways related)

Term ID	Term	Gene ratio	*P* value	Count
GO:0005126	cytokine receptor binding	0.088	5.30E-18	24
GO:0005125	cytokine activity	0.079	8.01E-12	17
GO:0045236	CXCR chemokine receptor binding	0.313	2.88E-06	5
GO:0042379	chemokine receptor binding	0.111	4.20E-06	7
GO:0032813	tumor necrosis factor receptor superfamily binding	0.130	1.18E-05	6
GO:0008009	chemokine activity	0.125	1.45E-05	6
GO:0005149	interleukin-1 receptor binding	0.222	8.93E-05	4
GO:0005164	tumor necrosis factor receptor binding	0.129	0.00047	4
GO:0048248	CXCR3 chemokine receptor binding	0.400	0.00400	2
GO:0042056	chemoattractant activity	0.091	0.00650	3
GO:0004955	prostaglandin receptor activity	0.200	0.00990	2

B. Cellular component enrichment (top 20 of related pathways)

Term ID	Term	Gene ratio	*P* value	Count
GO:0005615	extracellular space	0.041	1.18E-22	46
GO:0044421	extracellular region part	0.035	2.04E-21	48
GO:0005576	extracellular region	0.023	1.25E-17	57
GO:0045121	membrane raft	0.063	1.03E-11	19
GO:0012505	endomembrane system	0.014	3.17E-10	62
GO:0044444	cytoplasmic part	0.010	2.55E-09	94
GO:0031982	vesicle	0.018	3.64E-09	42
GO:0031410	cytoplasmic vesicle	0.018	1.43E-08	40
GO:0031983	vesicle lumen	0.047	2.37E-08	16
GO:0034774	secretory granule lumen	0.046	8.85E-08	15
GO:0098805	whole membrane	0.020	0.00000016	31
GO:0070013	intracellular organelle lumen	0.012	0.000000223	62
GO:0030141	secretory granule	0.027	0.00000024	22
GO:0005783	endoplasmic reticulum	0.018	0.000000246	33
GO:0044433	cytoplasmic vesicle part	0.020	0.000000358	29
GO:0099503	secretory vesicle	0.024	0.000000475	23
GO:0005901	caveola	0.108	0.000000734	8
GO:0005737	cytoplasm	0.009	0.00000421	97
GO:0031012	extracellular matrix	0.042	0.00000475	12
GO:0044446	intracellular organelle part	0.009	0.00000614	83

C. Biological process enrichment (top 20 of related pathways)

Term ID	Term	Gene ratio	*P* value	Count
GO:0034097	response to cytokine	0.050	5.52E-31	52
GO:0002682	regulation of immune system process	0.039	2.63E-27	54
GO:0071345	cellular response to cytokine stimulus	0.048	2.00E-26	46
GO:0019221	cytokine-mediated signaling pathway	0.061	3.70E-26	40
GO:0006954	inflammatory response	0.068	9.92E-23	33
GO:0002684	positive regulation of immune system process	0.045	9.76E-22	40
GO:0002376	immune system process	0.024	4.35E-20	58
GO:0043408	regulation of MAPK cascade	0.048	6.41E-19	34
GO:0043410	positive regulation of MAPK cascade	0.059	7.42E-19	30
GO:0030155	regulation of cell adhesion	0.050	1.24E-17	31
GO:0050727	regulation of inflammatory response	0.071	1.05E-16	24
GO:0022407	regulation of cell-cell adhesion	0.063	5.83E-15	23
GO:0050900	leukocyte migration	0.071	1.33E-14	21
GO:0001817	regulation of cytokine production	0.044	4.69E-14	27
GO:1903037	regulation of leukocyte cell-cell adhesion	0.072	5.09E-14	20
GO:0050776	regulation of immune response	0.036	7.46E-14	31
GO:0002694	regulation of leukocyte activation	0.051	8.67E-14	24
GO:0045785	positive regulation of cell adhesion	0.059	9.28E-14	22
GO:0006955	immune response	0.025	5.94E-13	39
GO:0002685	regulation of leukocyte migration	0.091	1.07E-12	16

Table S3. KEGG enrichment of Baimai ointment in the treatment of osteoarthritis

**Term ID**	**Term**	**Gene ratio**	***P* value**	**Count**
hsa05200	Pathways in cancer	0.082	5.42E-32	42
hsa04933	AGE-RAGE signaling pathway in diabetic complications	0.255	2.65E-29	25
hsa04668	TNF signaling pathway	0.167	5.85E-18	18
hsa04657	IL-17 signaling pathway	0.185	1.04E-17	17
hsa05161	Hepatitis B	0.134	1.04E-17	19
hsa05212	Pancreatic cancer	0.216	1.04E-17	16
hsa05219	Bladder cancer	0.341	1.04E-17	14
hsa05215	Prostate cancer	0.175	1.15E-17	17
hsa05418	Fluid shear stress and atherosclerosis	0.135	5.57E-17	18
hsa05142	Chagas disease (American trypanosomiasis)	0.158	4.52E-16	16
hsa04926	Relaxin signaling pathway	0.131	7.16E-16	17
hsa05206	MicroRNAs in cancer	0.114	5.27E-15	17
hsa05167	Kaposi's sarcoma-associated herpesvirus infection	0.098	6.76E-15	18
hsa05205	Proteoglycans in cancer	0.092	1.76E-14	18
hsa05144	Malaria	0.255	2.38E-14	12
hsa05134	Legionellosis	0.222	9.46E-14	12
hsa04060	Cytokine-cytokine receptor interaction	0.072	1.36E-13	19
hsa04218	Cellular senescence	0.103	1.36E-13	16
hsa05225	Hepatocellular carcinoma	0.098	2.29E-13	16
hsa05321	Inflammatory bowel disease (IBD)	0.194	3.24E-13	12
hsa05145	Toxoplasmosis	0.128	3.27E-13	14
hsa05323	Rheumatoid arthritis	0.155	3.27E-13	13
hsa04064	NF-kappa B signaling pathway	0.140	9.73E-13	13
hsa05143	African trypanosomiasis	0.294	1.10E-12	10
hsa04066	HIF-1 signaling pathway	0.133	1.65E-12	13
hsa04620	Toll-like receptor signaling pathway	0.127	2.52E-12	13
hsa05164	Influenza A	0.089	3.97E-12	15
hsa04151	PI3K-Akt signaling pathway	0.055	9.76E-12	19
hsa05226	Gastric cancer	0.095	1.03E-11	14
hsa05223	Non-small cell lung cancer	0.167	1.14E-11	11
hsa05146	Amoebiasis	0.128	1.77E-11	12
hsa05140	Leishmaniasis	0.157	1.89E-11	11
hsa05165	Human papillomavirus infection	0.057	1.89E-11	18
hsa05133	Pertussis	0.149	3.08E-11	11
hsa05152	Tuberculosis	0.081	6.12E-11	14
hsa05210	Colorectal cancer	0.129	1.14E-10	11
hsa05222	Small cell lung cancer	0.120	2.41E-10	11
hsa04115	p53 signaling pathway	0.147	2.95E-10	10
hsa01522	Endocrine resistance	0.116	3.14E-10	11

hsa01524	Platinum drug resistance	0.143	3.62E-10	10
hsa04068	FoxO signaling pathway	0.092	4.40E-10	12
hsa05160	Hepatitis C	0.092	4.66E-10	12
hsa05166	HTLV-I infection	0.060	5.08E-10	15
hsa04659	Th17 cell differentiation	0.108	5.61E-10	11
hsa04210	Apoptosis	0.089	6.02E-10	12
hsa05220	Chronic myeloid leukemia	0.132	6.57E-10	10
hsa05224	Breast cancer	0.082	1.44E-09	12
hsa04010	MAPK signaling pathway	0.051	3.69E-09	15
hsa04621	NOD-like receptor signaling pathway	0.072	5.08E-09	12
hsa05214	Glioma	0.132	5.08E-09	9
hsa05218	Melanoma	0.125	7.77E-09	9
hsa05014	Amyotrophic lateral sclerosis (ALS)	0.160	1.08E-08	8
hsa05168	Herpes simplex infection	0.066	1.18E-08	12
hsa05132	Salmonella infection	0.107	2.54E-08	9
hsa05213	Endometrial cancer	0.138	2.95E-08	8
hsa04110	Cell cycle	0.081	4.03E-08	10
hsa05202	Transcriptional misregulation in cancer	0.065	6.20E-08	11
hsa04371	Apelin signaling pathway	0.075	7.82E-08	10
hsa04915	Estrogen signaling pathway	0.075	7.82E-08	10
hsa04672	Intestinal immune network for IgA production	0.159	1.00E-07	7
hsa04062	Chemokine signaling pathway	0.061	1.13E-07	11
hsa04932	Non-alcoholic fatty liver disease (NAFLD)	0.067	2.01E-07	10
hsa01521	EGFR tyrosine kinase inhibitor resistance	0.103	2.14E-07	8
hsa04726	Serotonergic synapse	0.080	2.18E-07	9
hsa04630	Jak-STAT signaling pathway	0.063	3.60E-07	10
hsa05416	Viral myocarditis	0.125	4.10E-07	7
hsa04540	Gap junction	0.092	4.41E-07	8
hsa04380	Osteoclast differentiation	0.073	4.64E-07	9
hsa05332	Graft-versus-host disease	0.167	7.00E-07	6
hsa05162	Measles	0.068	7.90E-07	9
hsa04510	Focal adhesion	0.051	2.05E-06	10
hsa04919	Thyroid hormone signaling pathway	0.070	3.01E-06	8
hsa04020	Calcium signaling pathway	0.050	7.96E-06	9
hsa04215	Apoptosis - multiple species	0.161	8.12E-06	5
hsa04370	VEGF signaling pathway	0.102	9.03E-06	6
hsa05203	Viral carcinogenesis	0.049	9.09E-06	9
hsa05020	Prion diseases	0.152	1.03E-05	5
hsa04660	T cell receptor signaling pathway	0.071	1.23E-05	7
hsa05330	Allograft rejection	0.143	1.30E-05	5
hsa05216	Thyroid cancer	0.135	1.65E-05	5
hsa04015	Rap1 signaling pathway	0.044	1.91E-05	9
hsa04217	Necroptosis	0.052	2.17E-05	8
hsa04940	Type I diabetes mellitus	0.125	2.24E-05	5

hsa04670	Leukocyte transendothelial migration	0.063	2.45E-05	7
hsa04071	Sphingolipid signaling pathway	0.060	3.01E-05	7
hsa05204	Chemical carcinogenesis	0.079	3.07E-05	6
hsa05010	Alzheimer's disease	0.048	3.58E-05	8
hsa04014	Ras signaling pathway	0.039	4.29E-05	9
hsa04012	ErbB signaling pathway	0.072	4.75E-05	6
hsa04350	TGF-beta signaling pathway	0.072	4.75E-05	6
hsa04912	GnRH signaling pathway	0.068	6.34E-05	6
hsa05169	Epstein-Barr virus infection	0.041	9.10E-05	8
hsa00590	Arachidonic acid metabolism	0.082	0.00013	5
hsa04921	Oxytocin signaling pathway	0.047	0.00013	7
hsa04080	Neuroactive ligand-receptor interaction	0.033	0.00015	9
hsa04934	Cushing's syndrome	0.046	0.00015	7
hsa04931	Insulin resistance	0.056	0.00017	6
hsa04917	Prolactin signaling pathway	0.072	0.00022	5
hsa00980	Metabolism of xenobiotics by cytochrome P450	0.071	0.00023	5
hsa04622	RIG-I-like receptor signaling pathway	0.071	0.00023	5
hsa04520	Adherens junction	0.070	0.00024	5
hsa00983	Drug metabolism - other enzymes	0.066	0.00032	5
hsa04650	Natural killer cell mediated cytotoxicity	0.048	0.00034	6
hsa04658	Th1 and Th2 cell differentiation	0.057	0.00060	5
hsa00330	Arginine and proline metabolism	0.083	0.00064	4
hsa04750	Inflammatory mediator regulation of TRP channels	0.054	0.00072	5
hsa00220	Arginine biosynthesis	0.150	0.00078	3
hsa04923	Regulation of lipolysis in adipocytes	0.075	0.00088	4
hsa04623	Cytosolic DNA-sensing pathway	0.065	0.00150	4
hsa05230	Central carbon metabolism in cancer	0.062	0.00180	4
hsa05310	Asthma	0.107	0.00180	3
hsa00982	Drug metabolism - cytochrome P450	0.061	0.00190	4
hsa04114	Oocyte meiosis	0.043	0.00190	5
hsa04664	Fc epsilon RI signaling pathway	0.060	0.00190	4
hsa04722	Neurotrophin signaling pathway	0.043	0.00190	5
hsa05120	Epithelial cell signaling in Helicobacter pylori infection	0.061	0.00190	4
hsa05211	Renal cell carcinoma	0.059	0.00200	4
hsa01523	Antifolate resistance	0.097	0.00230	3
hsa04611	Platelet activation	0.041	0.00230	5
